# Vaccination in the Light of the Royal British Commission

**Published:** 1897-10

**Authors:** M. R. Leverson

**Affiliations:** Fort Hamilton, L. I., N. Y.


### VACCINATION IN THE LIGHT OF THE ROYAL BRITISH COMMISSION.

M. R. LEVERSON, M. D., FORT HAMILTON, L. I., N. Y.

(Continued from page 360.)

Q. 900. "You have not ascertained these deaths of unvaccinated persons from small-pox for such years as they are available, with a view to test that question?" "I personally have not."

At page 78, *supra*, an extract was given from Florence Nightingale's notes on nursing, and (Q. 901) Dr. Collins asks Dr. Thorne what is his view of her statement. He replies: "I am sorry to say that my own view would be very much what I said on the last occasion, that you might as well talk of a horse turning into a dog."

Q. 912. By Mr. Picton. "In answer to Q. 744, you spoke of the effectiveness of vaccination being in part dependent upon the number of scars; is the effect of vaccination in the body entirely local; that is to say, does it only extend to the part affected by the immediate pustule?" "I imagine that a patient who has one or more pustules is suffering from an acute specific disease, namely, cow-pox, which affects the whole system."

Q. 913. "Would the same amount of matter put into one incision have less effect on the system than if it were put into four incisions?" "I am not prepared to answer the question; it is a very difficult one to answer." And when the question is put more pointedly, with reference to the disease of cow-pox, whether it would be made more severe in one case or the other (Q. 916), he answers: "I am not prepared to differentiate between the local and general effect on that point."

Q. 915. "Is it not supposed to have a spreading effect upon the blood?" "*We really know absolutely nothing as to the effect of the specific poisons upon the blood.*"

(And yet the vaccinators pour them recklessly into it.)

Q. 916. "Do you not believe that cow-pox is self-multiplying when once inserted in the body?" "I am not prepared to answer that as regards cow-pox."

In answer to Q. 918, he says: "We attach the greatest importance to the quality of the lymph, in so *far as it can be judged of.*"

Q. 919. "How can you judge of it?" "*By the local effects* that it has produced upon those from whom it was taken."

Q. 920. " Has not some lymph a genealogy which shows it to have produced different effects upon different people?" "It might do so, but I could not say that it would be the lymph alone that did it. The same lymph will produce different effects upon two different children, according as those two different children are in a different state of health, and are exposed to different circumstances."

Q. 921. "Are you able by the microscope, or by any other mode of investigation, to find any difference between one lymph and another, apart from the symptoms induced by its insertion?" Ans. "I am no skilled microscopist, but, speaking generally of what is termed good lymph, I believe there is no difference detectable under a microscope."

Q. 922. "Have you yourself examined it under a microscope?" "I am no microscopist."

Q. 927. "I understood you to say that they (insanitary conditions) could not have much influence upon the spread of small-pox. Am I right in that?" "I believe that, apart from that one influence of crowding together, either on area or within dwellings, the *influence of sanitary circumstances is so insignificant that it is hardly worth naming in connection with the subject that is now before the Commission.*"

Q. 930. "You would allow some effect to sanitary conditions?" Ans. "The truth is this: That if sanitary conditions, apart from the ones I have named, have any influence upon small-pox, it is so absolutely overwhelmed and marked by the influence of vaccination, that it is not apparent to my mind."

Q. 934. "In answer to Q. 764, you said: 'Dr. Buchanan has shown that in London the saving of life from small-pox amongst infants has been greater amongst children getting their primary vaccination at public vaccination stations than amongst those who received their vaccination at the hands of private practitioners.' I suppose I should be right in interpreting that as equivalent to saying that the operation, as carried out at public stations, has been more effective in preventing small-pox than the operation as carried out by private practitioners?" Ans. "*That is so.*"

Q. 935. "I suppose you have seen the curves drawn out by Dr. Alfred Russel Wallace?" "*I never read his book or his pamphlet.*"

[Dr. Wallace is the man who, though his name is not so popularly known as that of Darwin, shares with him to the full the merit of discovering and elucidating the theory of evolution, and among men of science stands certainly not lower, and, some think, even higher than that profound and exact naturalist.]

Q. 938. "Between 1862 and 1863, did the number of official vaccinations rise considerably?" "The number rose between 1862 and 1865 from 437,693 to 578,583."

Q. 938a. "Would you kindly give me the number from 1862 to 1863?" "I am quoting from the book that you have handed me."

Q. 939. "But it is an official book?" "True. I am only doing it in answer to your question; I am not putting in any evidence upon this point. In 1862 there were 437,693 persons successfully vaccinated at the expense of the poor rates; the corresponding number for the subsequent year, 1863, was 646,464."

Q. 940. "That is a considerable increase, is it not?" "It is an increase."

Q. 941. "Is it the case that small-pox increased that year in 1862-63?" "I have not the figures before me."

Then follow Q. 943-964, a strange struggle on the part of Dr. Thorne to avoid giving evidence as to the increase of small-pox, which Mr. Picton (Q. 943) shows him seems to follow every increase in official vaccinations, while small-pox decreases as the official vaccinations decrease. He even struggles against being bound by a table (App. IV, pp. 122-3) handed in by himself on the request of Lord Herschell and Mr. Picton, and prepared by a clerk in the office of the Local Government Board, and with which he wrote to the Secretary of the Commission, "Herewith I enclose a table showing what we have been able to do in order to comply with the request made to me by Lord Herschell." (Q. 950.)

Q. 969. Mr. Bradlaugh obtains from him the statement that he objected to answer some questions put by Dr. Collins, on the ground that he "could hardly give evidence to the Commission of any value upon the past history of vaccination," and Q. 970 calls his attention to the fact that, in answer to the Chairman, he had made many statements relating to the past history of vaccination.

Q. 971. "Would your own statement in qualification apply to those?" "No, not altogether; I endeavored to give certain facts affecting the history of vaccination, in so far as those facts had influenced the attitude of the Medical Department of the Local Government Board toward this question of vaccination."

Q. 975. "You think it would be unfair to compare deaths among the unvaccinated anterior to vaccination with the deaths amongst the unvaccinated since vaccination has been enforced?" "My difficulty would be that you select one single town, and I do not know anything about the circumstances that governed either the small-pox or any other fever or disease some 200 to 300 years ago."

Q. 976. Asked if that would not be equally true with regard to statistics in pre-vaccination days used before the Commission, he replies: "I quoted them in so far as they support the general body of evidence that in pre-vaccination days the main incidence of small-pox was upon the infantile population."

Q. 977. " Do I understand that the value of any particular state of facts before this Commission is dependent upon whether or not they support some particular opinion?" "I have not desired to convey that impression; if you infer it from what I have said, I must have expressed myself badly."

Q. 980. "Does the virulence of small-pox affect the percentage under the age of fifteen of the total deaths?" "I do not think I am quite prepared to answer that question."

Qs. 981-2. He knows that only a very small percentage of children born in Sheffield remain unvaccinated, as regards recent births, but refers the Commission to Dr. Barry, on the subject of his report.

Q. 994. By Mr. Savoey. Relates to the "Preussen." Ans. "I have some more information I could give, since special attention appears to have been directed to this point. The Local Government Board in 1886 *took some pains to get the figures of the steamship 'Preussen,'* bound for Australia, on board of which small-pox broke out. You have, of course, on a vessel people living under the same sanitary circumstances, eating very much the same food, and in all respects practically alike, with the one solitary exception of vaccination. There were 312 persons on board this vessel. Of persons both vaccinated and revaccinated, there were 55. Four of these were attacked by small-pox; none died. Of persons vaccinated, but not revaccinated, there were 209; 45 of whom were attacked by small-pox, and 3 died; 13 persons had previously had small-pox, of whom 3 were attacked by small-pox and none died. Of persons stated to be vaccinated, but showing no scars, there were 16, two of whom were attacked by small-pox, and none died. Lastly, there were 19 persons unvaccinated; 15 of these were attacked by small-pox, and 9 died. This evidence is in expansion of that I gave showing that sanitary circumstances have little or no control over small-pox, when compared with the condition of vaccination or no vaccination."

It will be as well to mention here that it was afterward proved that the "Preussen" had 721 persons on board, instead of only 312, as stated by Dr. Thorne. Her crew consisted of 123 persons, including three stewardesses. Of the steerage passengers, 544 in number, 392 were British, who *were taken to Antwerp*, to be there embarked on board the "Preussen," to avoid compliance with the British Acts of 1855 and 1860, which insure something like decency and sanitation for emigrants. At Southampton, 16 cabin passengers were taken aboard, and on November 7th she sailed for Port Saïd, where, on the 18th, 35 additional passengers were taken on board, and the vessel was there delayed until the 22d, to await the arrival of the mails *via* Brindisi. Leaving Port Saïd on the 22d, the ship reached Aden on the 27th, having taken on 5 more passengers at Suez. This makes 721 persons, and yet Dr. Thorne says "the Local Government Board was at some pains to get the figures."

It will be further seen, when we come to the testimony of Dr. MacLaurin, that the ship was in a most filthy condition; that the discipline was bad, the smell abominable, and the dirtiest ship the quarantine officer at New South Wales had ever seen! That the cubic space allowed for the passengers was less than that allowed in a very crowded line of battleship in the old days of the British navy; that the number of closets was wholly insufficient; that there were 14 cases of small-pox among the crew, all of whom had been vaccinated, and some revaccinated, six of them revaccinated more than once.

Q. 995-6. After seeking to show that the small-pox deathrate of Leicester was less *before* any sanitary works were carried out than during their construction, 1856 to 1861, and subsequently 1862 to 1864, Mr. Picton asks, Q. 997: "You remember the great epidemic of 1872 and 1873 in Leicester, when a large number died of small-pox; are you aware that the spread of that epidemic was largely attributed by the inhabitants to the unsanitary condition of Leicester in those years, long after the dates you have referred to?" "*I cannot say that I ever* heard that allegation."

Q. 998. "Are you aware that the authorities immediately set to work to improve the sanitary condition of the town, which they considered grossly unsatisfactory?" "I know that Leicester has done a large amount of good sanitary work since that date."

Q. 999. "Are you aware that since they began those thorough improvements the rate of small-pox in Leicester has been exceedingly low?" "I know it has been low in Leicester, and in a very large number of towns since that date."

Q. 1,003. "Is it known to medical science in what way an attack of small-pox operates in preventing or diminishing susceptibility to subsequent infection?" "I think I can answer that nothing of any particular value is known upon that point."

Q. 1,009. "There were nine deaths during each of the last two years from small-pox in London."

Q. 1,010. "Judging from previous experience, we rather did anticipate that small-pox might by now have become more rife; and we cannot help thinking that the action which has been taken under your direction (Sir Edwin H. Galsworthy) of removing small-pox patients away from London, has probably tended to prevent that."

Q. 1,012. "By isolation?" "Yes, that has at least been one of the factors lessening it."

Dr. Thorne was recalled and examined on the 20th of

November, 1889. Qs. 3,823-41 describe the details of working the vaccination Acts. Q. 3,841 is of value, as it gives the instructions or orders of the Local Government Board, and is of interest to us as showing an attempt, at least, of securing some method, order, and care for the patient in England, and presents a marked contrast to the conduct of the boards of health in this country, in those respects. (See Appendix IX, pp. 380, 52c Rep. R. B. C.) In these instructions care is taken, or at least pretended to be taken, to secure an accurate record of every operation and its results.

It will doubtless astonish nine out of every ten vaccinating officials in the United States, to learn that the first of the instructions for vaccinators is the following:

(1.) "Except so far as any immediate danger of small-pox may require, revaccinate only subjects who are in good health. As regards infants, ascertain that there is not any febrile state, nor any irritation of the bowels, nor any unhealthy state of skin; especially no chafing or eczema behind the ears, or in the groin, or elsewhere in folds of skin. Do not, except of necessity, vaccinate in cases where there has been recent exposure to the infection of measles or scarlatina, nor where erysipelas is prevailing in or about the place of residence." (Page 284b, *ubi supra.*)

(3.) Gives directions to be given to the parents of other persons having the vaccinee in charge.

(6.) "Consider yourself strictly responsible for the quality of whatever lymph you use or furnish for vaccination. Never either use or furnish lymph which has in it any, even the slightest admixture of blood." (Page 284b, *ubi supra.*)

(8.) "Scrupulously observe in your inspections every sign which tests the efficiency and purity of your lymph." (Page 285a, *ubi supra.*)

Q. 3,853. Dr. Thorne testifies that special inquiries are made of the Local Government Board, of allegations of injury from vaccination since 1882, but (Q. 3,857) the Local Government Board had not received any information touching two cases mentioned by Sir James Paget.

Q. 3,859. Have made inquiry into every case of complaint. One case of tetanus (Q. 3,855), mentioned to-day, "is the only case of death from that cause that we have heard of."

Q. 3,856. "But I meant those two cases that we have had before us to-day." "I do not know what they were."

Q. 3,860. Information comes to the Board, generally, either from the parent or a medical man.

Q. 3,862. "We have no means of hearing of any other cases, except when the Medical Inspector is on his visit."

Q. 3,866. Is not able to say how many come to the Local Government Board in the course of a year.

Q. 3,923. By Mr. Collins. "Are you prepared to give us information with regard to the current lymph in use at the different establishments?" "Mr. Farn would know more about that than I do."

Asked as to any one who could give information as to the original sources of the lymph (Qs. 3,924-5), he says: "I do not know any one who knows anything absolutely as to the original sources."

Q. 3,926. "Does the Local Government Board make no inquiry as to the source of new stocks of lymph which are introduced?" "Certainly, if the Local Government Board issues any new stocks, but it has issued no new stock, except when the calf-lymph station was established."

Q. 3,927. "Did they make inquiry into the nature of that lymph?" "As I explained in my previous evidence, I was not located at Whitehall at that time. I am not aware of the details of the steps that were taken when the calf-lymph station was established. I could not give you any proper information upon that point."

Q. 3,928. "Would Mr. Farn be able to tell us?" "I am afraid I cannot answer for Mr. Farn. Dr. Cory would be able to tell you."

Q. 3,929. He believes no new source has been introduced beyond that which was obtained in the institution of the calf-lymph establishment in Lamb's Conduit Street.

Q. 3,930. "No other source has been introduced at the calf-lymph establishment?" "I could not give you information about the source of the lymph at the calf-lymph station."

Q. 3,931. "In answer to Q. 805, in your previous evidence, you said: 'We have, as far as the Local Government Board is concerned, adhered absolutely to the original sources, namely, that at Lamb's Conduit Street, and the humanized source, from specially-selected public vaccination stations.'" "I know of no change. I think I have repeated that to-day."

Q. 3,932. "Do I understand you to be aware of no new introduction of lymph at the calf-lymph establishment within a recent period?" "I am afraid I ought not to give evidence upon this point. Now you mention it, I believe there was some new lymph introduced, but I have no information about the source of lymph at the calf-lymph station; therefore, I would rather you asked some one who knows more about it than I do myself."

Q. 3,933. "Dr. Cory would know about it?" "Yes."

But it will be seen (*post*, Q. 4,284, etc.) that he does not.

Q. 3,934. "Has the Local Government Board at any time used or distributed lymph which has been derived from the inoculation of a cow with small-pox?" "I am not aware that we have."

Q. 3,935. "Does the Board accept that as a source of vaccine lymph?" "I really do not know what the Board accepts; I have told you the only two lymphs they distribute."

Q. 3,936. "Have they not distributed any of Ceely's lymph, or Babcock's?" "I could not tell you."

Q. 3,937. "You do not know that they have not?" "I do not know that they have or that they have not. I do not know anything about it."

Questioned as to an inquiry instituted by the Local Government Board, in regard to certain injuries resulting from vaccination at Norwich, in 1882, he could remember nothing about it, but (Q.3,940) believes generally that the conclusion of the inspectors, Mr. Henly and Dr. Airy, that those cases of erysipelas following vaccination, some of which proved fatal, were due to some contamination of the lymph, which had escaped detection, but refers the Commission to Dr. Airy as the best witness thereon (Q. 3,941).

Q. 3,942. Knows that an outbreak of erysipelas followed vaccination at Gainsboro' some years ago, but that is all he can tell about it.

Q. 3,944. "Has the Local Government Board been able to trace the origin of erysipelas arising in connection with vaccination to any want of care in the way in which the act is administered?" "Speaking quite generally, I should say that the vast majority of cases of erysipelas following vaccination that come under our notice, are cases in which the erysipelas does not begin till certainly as late as the middle of the second week of vaccination, and then, according to our experience, it is more than doubtful whether the injury was due to the lymph at all; the probability being that it was due to the circumstance that some septic matter got access to a wound, which happened to be the wound produced by vaccination."*

Q. 3,945. "What instructions are issued with regard to the management of the vaccinated arm for the week or two following vaccination?" "We issue none, except as regards the use of the shield."

---

* If A stabs B and during the process of healing erysipelas sets in and the patient dies, A is generally said to be guilty of the death.

Q. 3,946. "Was the recommendation in favor of or against the employment of shields?" "Against the use of them."

Q. 3,947. "Is it considered preferable to leave the arm open?" "It is considered preferable to leave it open."

Q. 3,948. "Do you consider the presence of erysipelas at any time in the course of the maturation of the vaccine vesicles an integral part of the local manifestation of the disease?" *"No!"*

Q. 3,949. Are you aware that Dr. Jenner considered it to be so?" *"I am not,* and should think if he said so, he must have used the word erysipelas in a very different sense to that in which I should use it."

[See Jenner's *Inquiry*, pp. 7-8; Crookshank's *Reproduction*, in Vol. II of his *History and Pathology of Vaccination*, page 9, note*; also, Jenner's Letter to Pearson, 27th September, 1798; Barnes' *Life of Jenner*, in which he states: "The cow-pox inflammation is always of the erysipelas kind," and on other occasions he repeats this statement, and makes the presence of erysipelas a test of its being the, "real" and not the "spurious" kind.]

Q. 3,950. "Are you aware that on one occasion he obtained true vaccine lymph from an erysipelas swelling on the upper part of the thigh of a sucking calf?" "I have no special knowledge of the details of Dr. Jenner's investigations; I never heard of the case you speak of."

[Jenner's *Inquiry*, page 62; Crookshank's *Reproduction*, page 33.]

Q. 3,953. "Are there any means of ascertaining by examination of the lymph, microscopically or otherwise, whether in any case it is likely to occasion erysipelas or not?" *"I know of none."*

Q. 3,954. "Do you think that under any circumstances vaccination *per se* in the ordinary way in which it is carried out at public vaccination stations, has occasioned erysipelas?"

"I have no doubt that there have been cases in which erysipelas has followed vaccination, and has been actually due to the lymph used, but I more than doubt whether anything like the organism, which some people believe to be invariably associated with erysipelas, is introduced by means of the lymph itself."

Q. 3,956. "Are you aware of Dr. Pfeiffer's experiments with regard to that?" "I do not recall them at the moment."

Q. 3,960. "Can you tell me what was the first occasion on which repeated penalties were recovered under any Act?" "No, I cannot."

Q. 3,961. "Was it before the Act of 1871?" "I do not know."

Q. 3,962. "Has the Local Government Board issued any recommendations or advice or orders with regard to the vaccination of children in workhouses?" "Yes; it has issued a memorandum."

Q. 3,963. "Would you tell me what the recommendation was?" "The memorandum to which I refer was a circular issued by the Poor Law Department of the Local Government Board on the 27th of January, 1881, to boards of guardians in the metropolis, and it contained this statement: 'Some boards of guardians have passed a resolution requiring the medical officer, subject to the exercise of his judgment as to making an exception in particular cases, to secure the vaccination of all children born in the workhouse as soon as possible after birth, and it has been found practicable, as a rule, to vaccinate the children when six days old and to inspect the results on the thirteenth day, as the mothers in such cases rarely leave the workhouse within a fortnight after their confinement.' It is thus rather a statement of fact than a recommendation."

Q. 3,964. "Where such a practice has been adopted, has it been on the strength of that recommendation, or on account of the action of the guardians themselves?" "I have no doubt that in some cases it has been on the strength of this letter."

Q. 3,965. "Would it be true to say that 'the practice of vaccinating children shortly after birth was not adopted under any advice of the Local Government Board?' " "I am not quite certain that I follow you; are you speaking of a date before the issue of the letter, or after?"

Q. 3,966. "I am speaking of the present time. Would it be correct to say that the practice of vaccinating infants almost immediately after birth, or within the first week of life, was not initiated under any advice of the Local Government Board?" "The practice has been adopted in some instances, I have no doubt, on account of the issue of the Board's circular."

Q. 3,967. Dr. Thorne has no knowledge of guardians who are prosecuting, sitting on the bench as magistrates, except the statements in the daily papers.

Q. 3,970. Believes that by far the majority of instances of injuries following vaccination are due to what happens subsequently to the vaccination.

Q. 3,973. Speaking of verdicts by coroners' juries, he says: "There unquestionably have been cases in which vaccination has been set down as the cause of death."

Q. 3,974. Also injuries not fatal have been traced to vaccination.

Q. 3,975. "Do you happen to know of any instance in which an action has been instituted on account of malpractice against the vaccinator?" "I do not recall any such case."

Q. 3,977. Asked as to the storing of lymph, he says: "Mr. Farn would give you much more satisfactory evidence upon that point."

Q. 3,978. "And also as to precautions in avoiding the admixture of blood?" "Yes."

Q. 3,980. Dr. Thorne cannot state at what period subsequent to vaccination the responsibility of the public vaccinator terminates.

The foregoing lengthy extracts from Dr. Thorne's testimony justify, it seems to me, the severe criticism passed upon that gentleman in the January number of THE HOMŒOPATHIC PHYSICIAN. I think every reader will now be willing to accept my abstracts of the testimony as fair. I shall not, therefore, deem it necessary to do more than abstract the evidence in future, except when desirable to present some specially striking or important statement in the very words of the witness.

POSTSCRIPT.—The student of these abstracts of the testimony before the Royal British Commission will have noticed a delicious bit of self-stultification on the part of Dr. Sweeting. In answer to Q. 3,751 (see page 299 of THE HOMŒOPATHIC PHYSICIAN for August), he said that he agreed with Dr. Gayton, "That the unvaccinated are drawn from a more neglected class"—*i. e.*, more liable to attack—but he did *not* agree with him "that that would necessarily raise their mortality from small-pox." A few minutes later (would it be as many as five?) at 3,757, he said: The two classes are "strictly comparable as regards *attack;* as regards progress and *course of diseases* (*i. e.*, fatality); undoubtedly there might be a difference according to the state of life and previous history of the patient; antecedent circumstances unfavorable to health might have some effect upon the *severity* of the disease" (*i. e.*, fatality again); "but in my opinion, would not have any effect upon the liability to attack!" And this is another of the most shining lights of vaccination, and of the medical profession in England, vying with Dr. Cory (another of such shining lights) in self-contradiction, but unlike Dr. Cory in this: that while Dr. Cory fell down and confessed his self-contradictions, it seems probable that Dr. Sweeting did not even see those committed by himself.

And with this example of medical ignorance in high places, we find some persons, even other than place-hunting doctors, clamoring for a "National Board of Health," to extinguish the little freedom left to us by the State Boards of Health, and to destroy the public health by raising periodical scares, and polluting the blood of the healthy by the inoculation of morbid poisons.

